# Algorithm for the Treatment of Tip Malformation Combining a Clinical Qualitative Assessment and Specific Closed-Rhinoplasty Techniques Based on Retrospective Analysis of Pellegrini’s Fellows 40 Years’ Experience

**DOI:** 10.1007/s00266-024-04310-9

**Published:** 2024-08-26

**Authors:** Alberto Scattolin, Luca D’Ascanio, Pier-Francesco Galzignato, Pietro De Luca, Niana Orlando, Massimo Ralli, Arianna Di Stadio

**Affiliations:** 1 “Valerio Micheli-Pellegrini” Study Center, “Villa Donatello” Clinic, Florence, Italy; 2 Department of Otolaryngology—Head and Neck Surgery, Civil Hospital, Fano, Pesaro Italy; 3 Otolaryngology Department, Isola Tiberina-Gemelli Isola Hospital, Rome, Italy; 4 Organ of Sense Department, University La Sapienza of Rome, Rome, Italy; 5 Otolaryngology Unit, Otolaryngology Department GF Ingrassia, University of Catania, Catania, Sicily Italy

**Keywords:** Rhinoplasty, Nasal tip deformities, Endonasal approach, Nasal cartilage, Tip grafts, Boxy tip

## Abstract

**Background:**

The aesthetic importance of nasal tip and the complexity of its surgical correction make the surgery of this area one of the most fascinating facial surgical procedures. Despite description of different sculpturing techniques to correct nasal tip defects, this surgery remains one of the most discussed and challenging aesthetic procedures.

**Objectives:**

The objective of this study was to define an algorithm of treatment for nasal tip surgery based on 40-year experience on Caucasian patients evaluated by our proposed clinical qualitative assessment, who were treated by closed rhinoplasty.

**Methods:**

We retrospectively reviewed 19,643 Caucasian patients (15,266 females and 4,377 males) who underwent primary closed rhinoplasty from 1979 to 2019 due to different tip defects. The patients were evaluated by volume projection rotation (VPR) assessment. The surgical indications options, long-term aesthetic results and complications were analysed.

**Results:**

22% patients with minimal nasal defects were treated by non-delivery approach and 78% patients with important tip malformation by delivery approach. In all cases, the surgery was performed to reduce tip volume and modify tip projection and rotation based on the specific nasal defects. 67% patients, who needed important reduction of tip projection, were treated by tip-interrupting techniques. 88.7% patients declared full satisfaction after surgery, and only 12.3% needed a requiring minor revision surgery during the 20 years follow-up.

**Conclusion:**

The proposed algorithm may be a useful tool to plan surgery. The use of an adequate technique depending on the evaluation of volume, projection and rotation may guarantee higher patients satisfaction and a stable long-term aesthetic result.

**Level of Evidence III:**

This journal requires that authors assign a level of evidence to each article. For a full description of these Evidence-Based Medicine ratings, please refer to the Table of Contents or the online Instructions to Authors www.springer.com/00266.

## Introduction

Rhinoplasty is considered one of the most complex procedures in aesthetic plastic surgery, and the surgery of the tip is considered as the most difficult and sometimes unpredictable phase. Because of the complexity of tip management, several corrections techniques have been proposed by the years [1–3]. Sheen, as first, used a pre-operative method to evaluate nasal malformation based on the use of angles, lines, and planes, especially at the level of the nasal tip. The ideal tip was defined as 2 equilateral geodesic triangles with a common base formed by a line connecting the 2 domes, creating a diamond shape [4]. Then, Toriumi introduced the concept of nasal tip contour defined by series of lights and shadows, in which the tip is represented by a horizontal tip highlight corresponding to its domes [5]. Recently, Çakir introduced a new concept of nasal and tip analysis based on the identification of polygons or geometric forms composed of lines, shadows, and highlights with specific breakpoints [6]. The correct definition of a method to evaluate the characteristics and the malformation of the nose is the first step for a correct planning of rhinoplasty and of the optimal surgical technique to use [4–6]. The best surgery aims at tissue preservation, reduced recovery time and best short- and long-term results [7].

Closed rhinoplasty is today an old-style approach to the nasal tip left behind by the open approach [8] that guarantees best management and clear view of the tip structures facilitating the procedure for the surgeon even in teaching context [8]. However, Rorhich and Afrooz in their recent review underlined that both open and closed rhinoplasty have strength and weakness and they should be used accordly to an exact evaluation of the needs based on correct evaluation of the nasal structures and pre-surgical planning [9]. In 2018, Daniel emphasized the benefit of using closed rhinoplasty by defining the technique as preservation rhinoplasty [10].

In this retrospective study, we present the results of a 40-year surgical experience using closed technique rhinoplasty; our patients were evaluated by a clinical assessment based on the qualitative analyses of the nasal tip such as volume (*V*), projection (*P*) and rotation (*R*).

Fellows of Valerio Pellegrini were instructed to use sequential techniques to improve the results of closed rhinoplasty and to use Pellegrini approaches and techniques.

This study aims to present the good and long-term satisfactory results obtained by combining a clinical qualitative assessment of nasal tip with specifical surgical techniques and to suggest based on the retrospective review of over 19,000 cases of closed rhinoplasty an algorithm to maximize the long-term results of tip surgery.

## Materials and Methods

We retrospectively analysed 19,643 Caucasian Mediterranean patients (15,266 females and 4,377 male) who underwent primary closed rhinoplasty between 1979 and 2019. Preoperative informed consent was obtained from all patients before surgery, including the authorization of using their data for research purpose. This study was performed in accordance with the Declaration of Helsinki and was authorized without release of a number due to its retrospective nature.

All patients were assessed by nasal endoscopy to evaluate the conditions of nasal septum and turbinates, and standard facial photographs (front, lateral, ¾, upper and basal views) were performed. Paranasal sinus computed tomography (CT) scans were performed when necessary. The assessment of nasal tip morphology, quality of skin texture and aesthetic results according to Byrd analysis was carried out by comparing pre- and post-operative volume, nasal length (distance between nasion and tip most projected point), tip projection (distance from a vertical facial plane passing through the alar crease to the nasal tip) and tip rotation (defined as the tip angle which is measured from the vertical plane at the alar crease to the tip) [3].

**Fig. 1:**
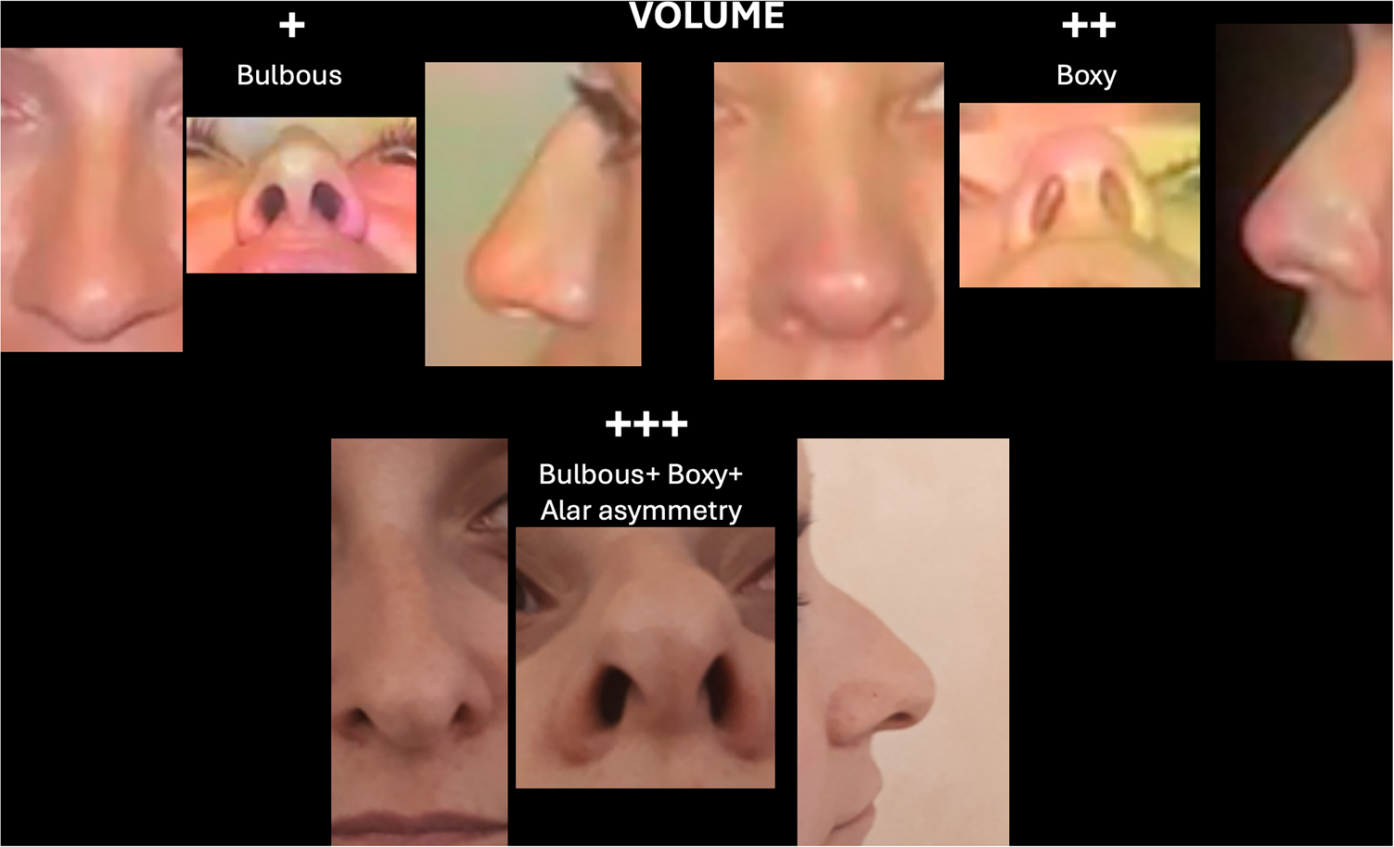
Example of assessment of volume using VPR assessment based on patient’s pictures

In the diagnostic phase, each parameter (volume, *V*; projection, *P*; rotation, *R*) was evaluated according to tip defects with variable degrees of severity defined as “+” (augmented) or “−” (reduced). Volume was considered “+” in case of bulbous tip, “++” in presence of boxy tip and “+++” when tip was bulbous, boxy and asymmetry of alar cartilage (Fig. 1). Negative sign which indicates reduced volume of the tip, was not considered because this malformation as primary condition is rare in Mediterranean population; in fact, reduced volume of the tip is generally secondary to previous nasal surgery [11].

**Fig. 2:**
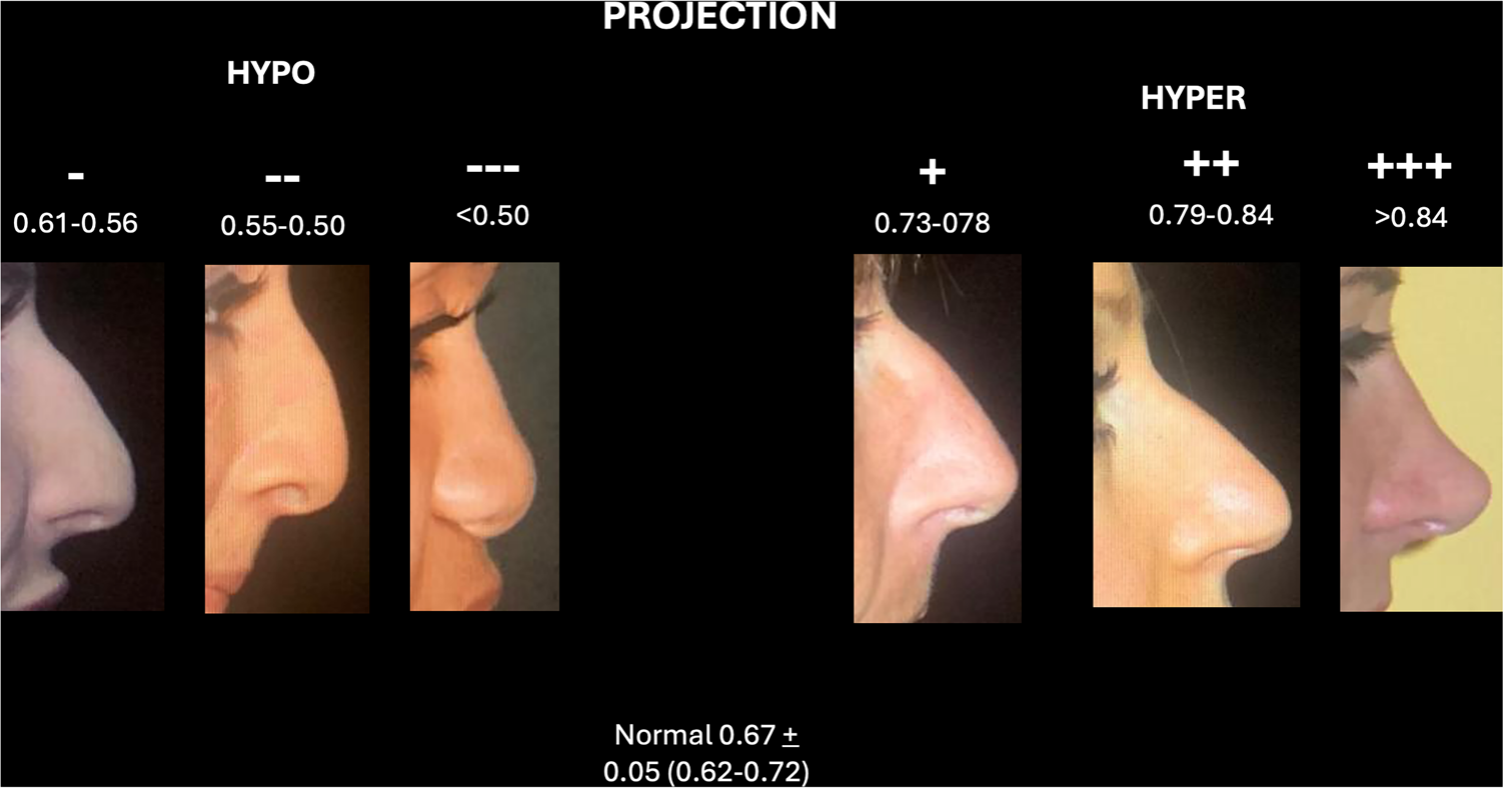
Example of the assessment of projection using VPR assessment. The evaluation includes both hypo- and hyper-projection

**Fig. 3:**
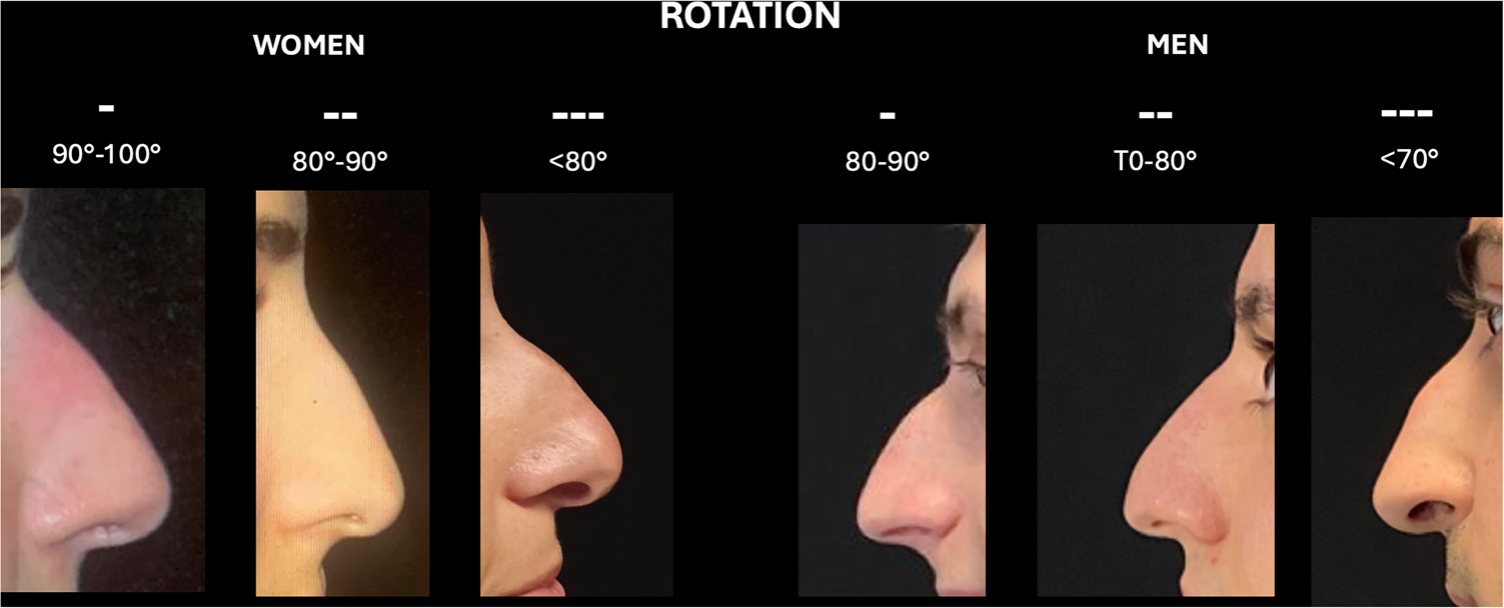
Example of assessment of rotation using VPR assessment. Because the standard angle is different for men (90°) and women (100°), this parameter must be evaluated by considering gender difference

Projection, nasal height divided by the nasal length, was documented as a percentage, with normal value 67 (±5). For value under this score was assigned a negative sign (−), for value over 67 was assigned a positive sign (−). The increase of signs number (-; --; --- or +; ++; +++) indicates the increase of severity (Fig. 2) [12]. Rotation was classified differently among gender because of normal standard (100° for women and 90° for men). This parameter has only negative score because typical Mediterranean nose are generally hyporotated [11] (Fig. 3).

To note, in case of normal parameter, i.e. volume, “0” was assigned in place of the mathematic sign (−/+), to define it.

Each tip defect was correlated, in the therapeutic phase, to a specific surgical modification as described in the literature [9, 10]. To simplify the diagnosis and planning, the acronym VPR was used for each clinical case to define the volume, projection and rotation parameters related to the therapeutic indications.

Figures 1, 2 and 3 show the details of VPR assessment.

Based on the severity of malformation we used specific techniques showed in Table 1 for volume, projection and rotations.

**Table 1:** The table summarizes the different used approach based of VPR evaluation

Tip volume reduction			Tip projection augmentation			Tip deprojection			Tip rotation		
Non-delivery approach	V+	LLCs cephalic trim	**Non-delivery approach**	P+	Umbrella graft		**P -**	Delivery approach			Retrograde or transcartilaginous incision
	V ++	LLCs cephalic trim		P ++	Transdomal suture			Anterior caudal septum resection	**Non-delivery approach**	**R +**	± Anterior caudal septum resection
Delivery approach		± Transdomal sutures	**Delivery approach**		± Columellar strut	**Delivery approach**	**P - -**	Transfixion Incision			Transfixion incision
		± Interdomal suture			± Umbrella graft			Anterior caudal septum resection		**R ++**	LLCs cephalic trim
	V +++	LLCs cephalic trim			± Lateral crura steal			Lateral crura overlay	**Delivery approach**		± Anterior caudal septum resection
		± DD or DA		P +++	DD		**P - - -**	DD or DA			Transfixion incision
					± Columellar strut			± Anterior Nasal Spine Resection		**R +++**	LLCs cephalic trim
					± Umbrella graft			Alar setback approach			± Anterior caudal septum resection
					± Shield graft (Sheen)						± ULCs caudal resection
											± DD
											± Pre-spinal graft
											±Tongue-in-Groove

Intra-, peri- and post-operatory complications were collected and reported when observed.

Three senior surgeons (AS, LDA, and PG) performed all the surgeries using intravenous sedation and local anaesthesia. Tip surgery was always performed using endonasal delivery or non-delivery (retrograde or transcartilaginous) approach according to the planned surgical tip modifications. We only used autologous grafts when necessary to support the nasal septum, or upper and lower lateral cartilages; resorbable sutures were used for tip nasal refinements. At the end of the procedure, nasal packing, sterile-strips, and a thermoplastic external nasal splint support were applied. Nasal packing was removed after 2 days and thermoplastic external nasal splint after 14 days. After cast removal, the follow-up was carried out 1, 3, 6, 12 and 24 months after surgery. At the same time, clinical evaluation and photographic study were performed.

Patients’ satisfaction was evaluated as described by Khamsa in 2016 [13]. The authors classified the patients who rated rhinoplasty as “worth it” were considered satisfied, and those who rated it as “not worth it” were considered as dissatisfied.

**Fig. 4:**
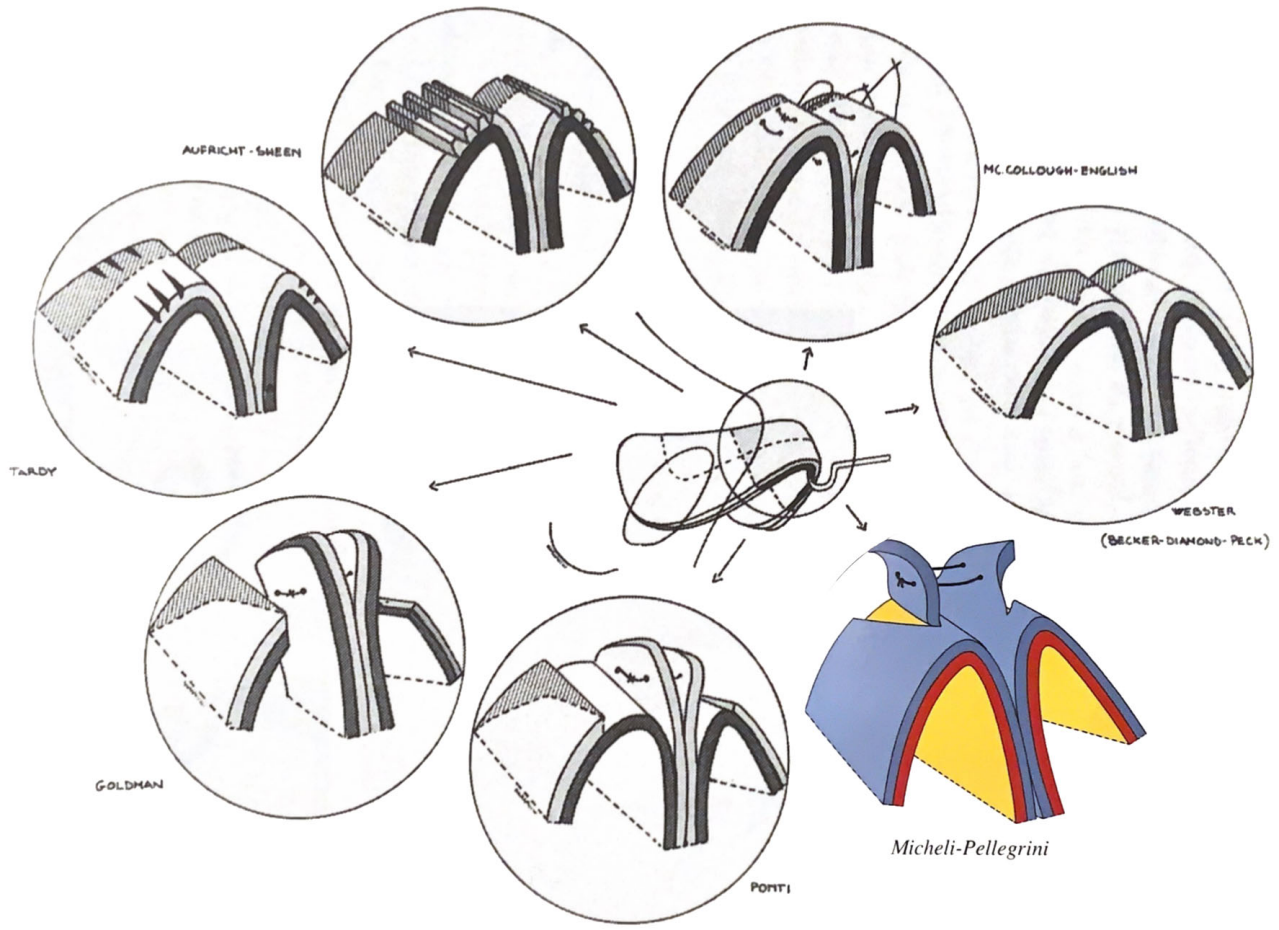
The coloured diagram shows the Valerio Micheli Pellegrini’s technique (VPT). The other technique than can be used and have been used by the time is represented by the black and white diagram. Compared to Ponti technique, VPT cuts and suture only the anterior portion of the alar cartilage without touching the rest. The preservation of the main body of the cartilage could be the key of long-standing satisfactory results

Surgery was performed following the sequential technique, a progressive step-by-step approach to the tip to improve the outcome defined by Valerio Micheli Pellegrini (Fig. 4).

As first step of the “sequential technique” parallel incisions of LLCs according to Aufricht’s and Tardy’s technique are performed that allows to reduce tip volume without changing its projection and rotation [14, 15]^.^ Then, a cartilage triangle is removed according to Becker’s technique to improve more tip definition and rotation [16]. As third the McCullough-English technique is used to reduce the domal and divergence angle, increase in tip projection and increase in tip rotation [17]. In conclusion, the dome division (DD) is performed; the division is done by preserving the inner mucosa lying [18] to improve the functional results of tip surgery. In case of severe malformation, we performed dome amputation (DA) associated when necessary to nasal grafts [16].

Table 1 shows the different surgeries that we performed based on the different VPR.

### Statistical Analyses

The satisfaction between patients was compared used Chi-square (*χ*) test, and the same test was used to compare the causes of revision surgery. Comparison between non-delivery versus delivery access was performed by Chi-square. Same test was used to compare interrupting technique versus dome amputation. We calculated the odds ratio (OR) for risk of failing (need of new intervention) comparing non-delivery versus delivery approach and DD versus DA. *P* was considered significant < 0.05. The tests were performed using Prisma 10 ®.

## Results

Table 2 shows the demographic information about our sample.

**Table 2:** Demographic characteristics of the sample

Patients	Gender	V			P			P			R		
		+	++	+++	+	++	+++	-	--	---	+	++	+++
19,643	Male	231	438	338	494	232	477	368	348	486	332	268	365
	Female	1572	1337	1019	1166	1167	1179	984	1187	949	2334	1667	1155

Among the 19.643, 14,339 (73%) patients performed a septo-rhinoplasty to restore normal nasal breath. None of the patients showed relevant comorbidities. In 100% cases, the surgical procedure was successfully completed without intra- or post-operative adverse event. 17,286 patients (88.3%) were satisfied of their aesthetic outcome and 2,416 subjects (12.3 %) were not. The latter underwent minor revision surgery (nasal tip bossae 1.9 %, pollibeak deformity 4.2 %, nasal tip ptosis 3.3 %, pinched tip 2.9 %) (Table 3). We used different approach for re-intervention depending on the specific problem. Pollibeak deformity was corrected by non-delivery independently from primary used approach, the others were treated by delivery and use of grafts when needed.

**Table 3:** The table shows the causes of re-intervention in our sample and the type of surgical technique used

	*Retrograde (%)*	*Transcartilaginous (%)*	*McCollough-English’s (%)*	LCS (%)	DD (%)	DA %	Total
Pollibeak	0,9	1,3	0,4	0,3	0,5	0,8	4,2
Nasal tip ptosis	0,4	0,3	0,4	0,7	0,7	0,8	3,3
Pinched tip	0	0	0,2	0	1,6	1,1	2,9
Nasal tip bossae	0,6	0,5	0,6	0,2	0	0	1,9
	1,9	2,1	1,6	1,2	2,8	2,7	**12,3**

In *italic* non-delivery approach, **bolded** delivery approach. The percentage refers to 12.3% only; so they are divided considering 12.3% as 100%

A statistically significant difference was observed between satisfied and non-satisfied patients (*χ*: *p* <0.0001); patients who were satisfied, as identified by the Khamsa questionnaire, were the majority.

**Fig. 5:**
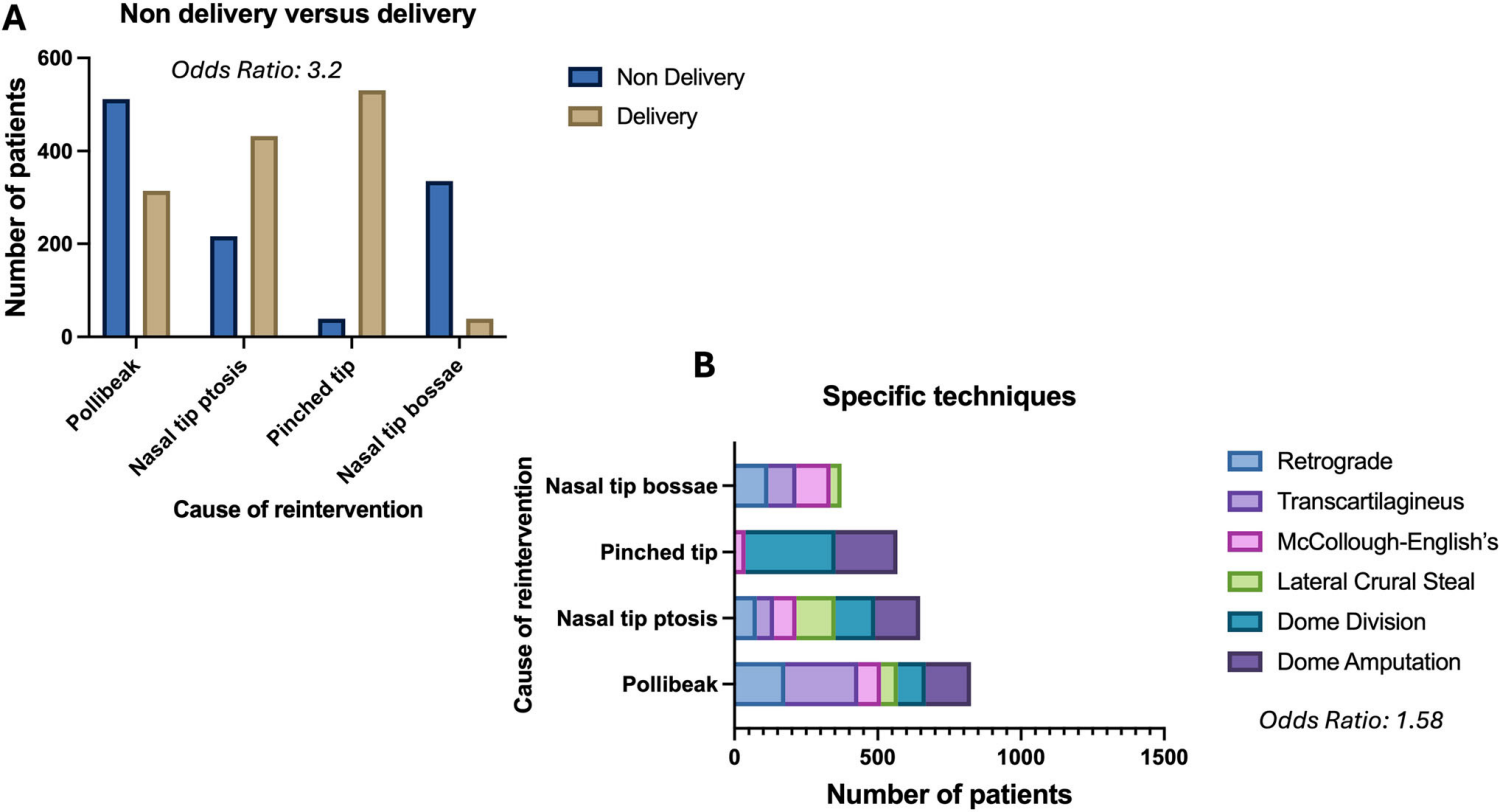
The graphs show the difference in the needs and the specific tip defect that cause re-intervention by using non-delivery and delivery approach (**A**) and within the specific techniques used (**B**)

Non-delivery approach (retrograde or transcartilaginous) was performed in 4.321 patients (22%) to correct V+, whether 15.322 (78%) with V++ and V+++ were treated by delivery approach for reducing tip volume and modifying tip projection (decrease (P -) or increase (P+)) and rotation (R+), depending on the type of nasal defects. The difference between the use of non-delivery versus delivery was statistically significant considering the need of re-intervention (*χ:* *p* <0.0001). Non-delivery approach was at higher risk of failing when compared to delivery approach (OR: 3.21; CI95: 2.93-3.51; *p *<0001) (Table 4 and Fig. 5A).

**Table 4:** The table shows the results of the study by summarizing them in delivery and non-delivery approach

	Approach	% patients	V	P		R
				Increasing	Decreasing	
	Retrograde	22	+		+	+
Non-delivery approach	Transcartilaginous	78	+		+	+
	McCollough-English’s	21	++	++	+/-	+
Delivery approach	Lateral Crura Steal	15	++	++	+/-	+
	Dome Division (DD)	53	+++	+++	+++	+++
	Dome Amputation (DA)	11	+++		+++	++

Among the 15.332 with V++ and V+++ , 67 % patients (10.265 people) underwent dome division (DD) or dome amputation (DA). The former was done in 61% of cases (6261 subjects) and the latter in 59% cases (4.004) . Statistically significant differences in the use of the two techniques, based on the patients’ needs, were identified (*χ*: *p* <0.0001). The risk of re-intervention was higher for patient who were treated by DA compared to the ones who underwent DD (OR: 1.58; CI95%: 1.39-179; *p *<0.0001). Statistically significant differences (*χ:* *p* <0.0001) comparing the 6 specific techniques Retrograde, Transcartilaginous, McCollough-English’s, Lateral Crura Steal, Dome Division and Dome Amputation and the needs of re-interventions based on the specific tip alterations were identified (Fig. 5B).

**Fig. 6:**
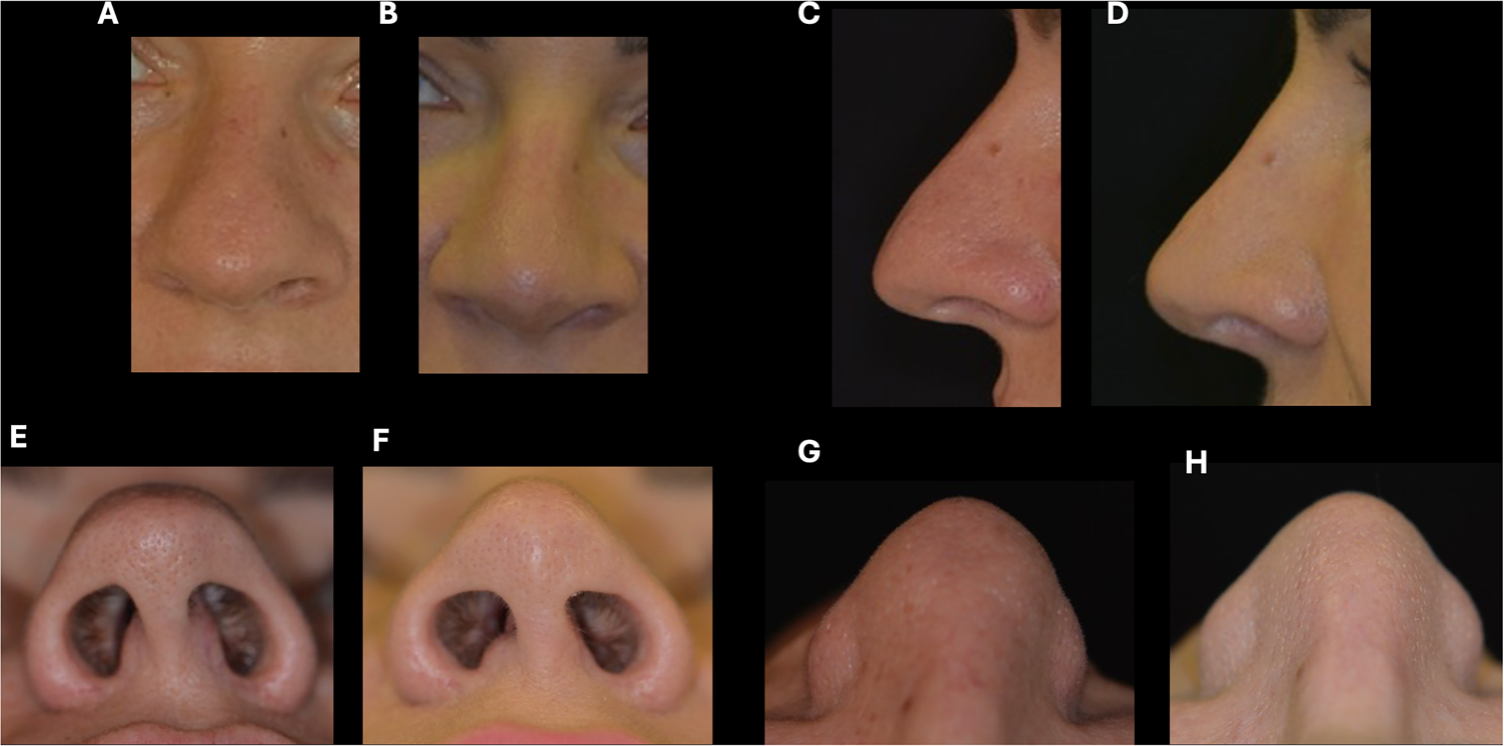
*V+++ P- R- Bulbous tip.* Pre (**A**, **C**, **E**,**G**) and 3-year postoperative (**B**, **D**, **F**,**H**) views of a 42-year-old female with tip deviation, large base and thick skin who underwent a closed rhinoplasty approach, dorsal hump reduction, cephalic trim excision of lower lateral cartilages, vertical dome division, tip/ graft and nasal osteotomies. Pre-surgery frontal view (**A**), pre-lateral view (**C**), pre-head tilted back view (**E**) pre-tip basal view (**G**). Post-surgery frontal (**B**), post-lateral view (**D**), post-head tilted back (**F**), post tip basal view from bottom (**H**)

**Fig. 7:**
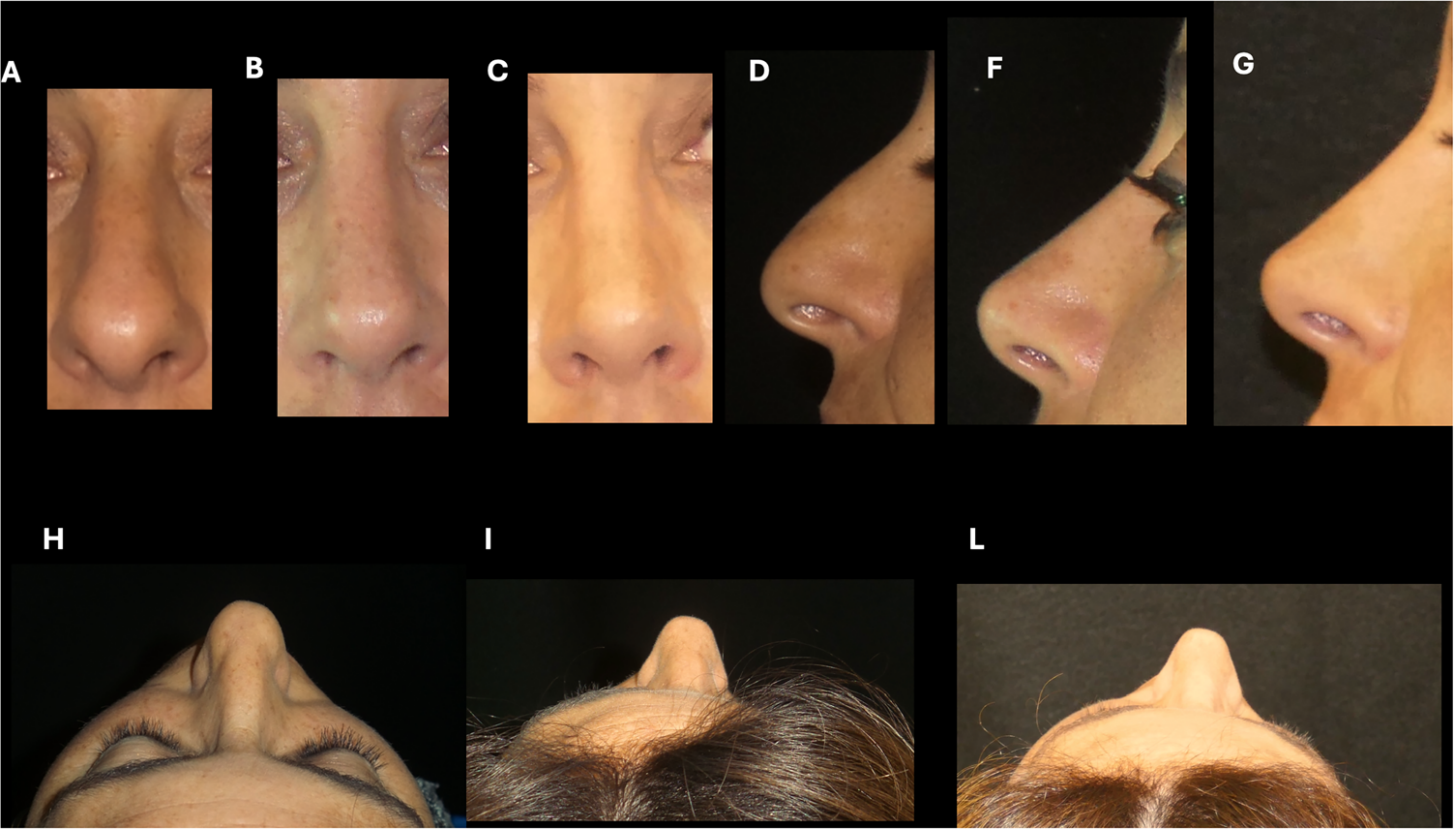
*V++ P- - R-. Reduction tip projection****.*** Preoperative (**A**,** D**,** H**), 1 year (**B**,** F**,** I**) and 3-year post-surgery (**C**,** G**,** L**) views of a 43-year-old female with increased tip projection who underwent an closed rhinoplasty approach, dorsal hump reduction, cephalic trim and margin reduction cephalic trim and margin reduction of lower lateral cartilages, vertical dome division, columellar strut placement and nasal osteotomies. Frontal view (**A**,** B**,** C**), lateral view (**D**,** F**,** G**), head tilted back view (**H**,** I**,** L**)

**Fig. 8:**
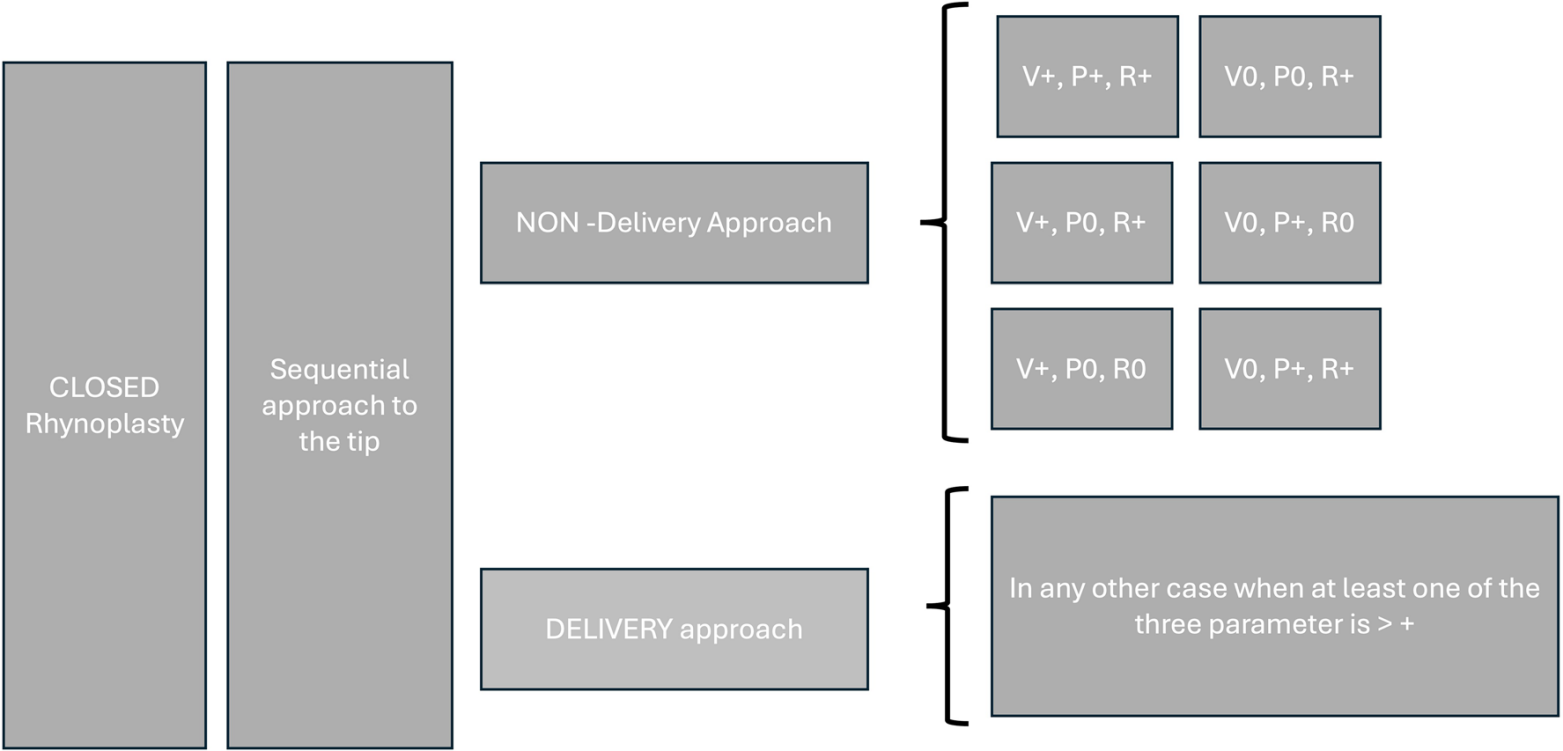
Based on the results of our study we defined the Valerio Micheli Pellegrini algorithm. The algorithm was defined based on the VPR scores

Figures 6,7 and 8 show some examples of our surgeries.

## Discussion

Our study showed that the use of correct surgical approach based on VPR assessment allowed to obtain statistically significant satisfaction by limiting the needs of revision surgery. The proper use of delivery or non-delivery approach was determinant to guarantee the best outcome. In most of the cases an interruption technique was necessary to correct the nasal tip; the use of interruption technique was always necessary in patients with high score in the VPR assessment. We observed that the use of non-delivery approach was associated with higher risk of re-intervention (OR:3.2); so, although the procedure is a little bit less traumatic than delivery, we suggest to use the delivery approach because it allows to perform tip surgery by direct visual observation reducing the re-intervetion risk.

Dome amputation was used less than dome division for correcting tip malformation; less use of DA was good because this technique had higher risk of re-operation compared to DD. We used DD to treat important alar malformation without using nasal graft, to obtain important increase in projection or to notably increase the rotation. We used DA to reduce tip projection or to reduce the surface in bulbous tip. In all cases, we used the sequential technique as described 40 years ago by Valerio Micheli Pellegrini.

We suggest that V+ should be treated by endonasal non-delivery approach (retrograde or transcartilaginous) and performing a minimal cephalic trim of LLCs. V++ needs delivery approach. In this case, a cephalic trimming of LLCs proportional to the tip volume reduction is requested, being careful to preserve minimal vertical height of 6 mm of alar cartilage to avoid any nasal valve collapse. In the presence of an interdomal divergence angle > 30, a single transdomal suture would be sufficient to define the dome anatomy, associated with an interdomal suture for gently close of the width of the tip, especially in female patients. In patients who present thick skin a tip cartilage "umbrella graft" (from the nasal septum), placed on the domal area and proportionally tailored is useful to better define the tip. To minimize the risk of graft displacement, it is mandatory to fix the domes with resorbable suture through the endonasal delivery approach. Finally, for more tip refinement, when a slight cephalic tip rotation required, the umbrella graft may be positioned more cranially.

For V+++ we always used delivery approach and the tip reduction is performed by cephalic trim of LLCs and transdomal sutures, often associated with an interdomal suture to improve the nasal tip definition. In patients with thick skin, the grafts (i.e. onlay, strut, shield or floating) are useful to improve the tip shape. The umbrella graft helps to add ulterior increase of the tip projection and/or avoid an over-narrow (“sting-like”) tip. In those patients with i) widened relatively broad domal arch and ii) wide nasal tip with a trapezoidal appearance on the base view (V++ and V +++), we combine DD/DA technique associated with columellar strut graft and umbrella graft. This may be a valid and useful technique to correct the abnormality of thick skin by narrowing the convergence of the apex and arch, increasing projection, and creating a better triangular base appearance.

To improve and increase tip projection it is possible to use both nasal tip suture and grafts [9, 14, 15].

We suggest using a non-delivery approach (retrograde or transcartilaginous incision) for P+ that needs a slight increase in tip projection. Furthermore, it is useful to associate a small tip graft (umbrella graft) and minimal lateral crura cephalic resection particularly in patients with thick skin [16]. To note, fixing the graft in a sub-SMAS pocket with resorbable suture guarantees stability and better outcome for long time.

In P++ we suggest a delivery approach, transdomal sutures associated with columellar strut graft and umbrella grafts particularly in patients with thick skin, exactly as for P+. An alternative could be lateral crura steal technique that can offer a better increase in tip projection [17, 18].

In P+++ the delivery approach is always suggested; then, DD technique associated with columellar strut and/or a shield graft and umbrella graft are useful to obtain great long-term stability and harmonic tip.

In some cases, patients need a decrease of their tip projection; in these cases, we recommend a non-delivery approach for minimal reduction (P-) combined with septal dorsal reduction to easy slightly lowering the tip. P-- needs the delivery approach to better manage the tip malformation; in these cases, we perform a transfixion incision to reduce the dorsal septum. In selected cases, it is useful to associate the lower lateral cartilages cross-hatching to ulteriorly reduce nasal tip projection. Finally, in case of P--- the delivery approach is always performed, and we use DD technique proportional to the extent of the planned tip deprojection to preserve the underlying skin, associated where needed, with anterior nasal spine reduction. In case of thick skin, it is useful to associate columellar strut insertion to support the new position of the tip. Such modification increases long-term stability of the result and avoids a post-operative pinched and over-narrow appearance of the nasal tip.

The nasal tip rotation (nasolabial angle that ranges in men from 90° to 105° and in women from 105° to 120°) as the other parameters (V and P) needs to be treated by specific techniques.

For this reason, we used a non-delivery technique in case of needing of slight cephalic tip rotation (R+); this approach was performed or retrograde or transcartilaginous incision. Then, we do minimally cephalic cut of LLCs and wedge caudal resection of the septum to slightly increase tip rotation. For R++ and R+++ we always perform an endonasal delivery approach. In case of R++ we do the cephalic resection of LLCs (bigger than in case of R+) and the wedge caudal resection of septum. For R+++ a transfixion incision is performed, and a substantial portion of the caudal septum is removed; then, LLCs are modelled in their cephalic and caudal portions to obtain better definition of the tip. Also, in these cases, the umbrella graft is placed in case of thick skin to better define the tip and improve the long-term results of the procedure.

Additional procedures can be performed in addition to DD (or as alternative in case of fail) like simple domal suturing, cephalic trim of LLCs, lateral crura overlay, and tongue-in-groove technique [9, 19]. DD should be associated with the resection of the caudal edge of the nasal septum and the border of the upper later cartilages to open the nasolabial angle and increase tip rotation. Another optional technique to increase tip rotation is the prespinal graft; this graft can be created by suturing together different cartilage fragments (sandwich style) by resorbable suture. By hemi-transfix incision it is possible to create a pocket to insert the graft; then, a transcutaneous suture is placed to fix the graft in the pocket [20].

In general, we use non-delivery approach in patients who need minimal corrections (V+, P+/P-, R+); if one of the three scores is higher (i.e ++) we always use delivery approach that allows to perform more complex techniques by preserving the tissue. Closed rhinoplasty for correction of tip malformation is a valid and safe procedure. It reduces the damage on the columella area improving the outcomes both a short and long term. Moreover, the closed approach allows to clearly understand the final aesthetic result intra-operatory, while the same is not possible by open technique.

Delivery or non-delivery approach is the real key of success as showed in our large sample of patients.

### Limitations of the Study

 This study presented showed major and minor limitations. A major limitation might be that we only used closed technique without comparison with open-currently considered as best surgical approach; however, we reported a wide sample of results that supported the use of closed rhinoplasty with delivery approach to obtain satisfactory long-term results. Although, today open rhinoplasty is the most used technique and innovative techniques are available to better define the tip [21], the closed technique can be still an option when a correct surgical planning is performed [9]. Another major limitation is the retrospective nature of the study. Moreover, because our follow-up was very long we did not analyze a series of confounders, that could have impacted on the results. The strength of a 20-years follow-up is also the weakness due to the different potential confounders that could have impacted on the outcome.

Additionally, the VPR assessment did not measure skin thickness in a rigorous manner, but skin is evaluated in the general context of the nose. This could be an important limitation of the assessment, especially because the skin thickness has a strong impact on the rhinoplasty outcomes. Finally, the algorithm that we proposed was based only on closed rhinoplasty techniques. Additional studies comparing different open and closed techniques are necessary to define a perfect algorithm; however, our proposal might be a starting point.

## Conclusions

Using a semi-qualitative assessment allowed us to correctly plan the surgery that allowed to obtain a satisfactory outcome in the long term. The delivery approach was less at risk of re-intervention because it allowed to better visualize and manage the tip cartilages. The DA, despite used in a minimal portion of our sample, showed higher risk of re-intervention due to bad outcome when compared to DD.

The sequential technique created by Valerio Micheli Pellegrini allowed a clear identification of what to do and respect the tissue for improving the outcome, especially because the used techniques were tailored on patient’s needs. The VPR assessment, despite less precise than millimetric pre-operatory measurements, could be a valid tool in expert hand surgeon to speed patients’ evaluation and plan the best techniques to use.

Finally, based on our results, we suggest that by following an algorithm in pre-operatory planning even by using closed rhinoplasty—considered an outdated technique—it may be possible to obtain harmonic, satisfactory, and stable results.
